# Regulation of catabolic gene expression in normal and degenerate human intervertebral disc cells: implications for the pathogenesis of intervertebral disc degeneration

**DOI:** 10.1186/ar2693

**Published:** 2009-05-12

**Authors:** S Jane Millward-Sadler, Patrick W Costello, Anthony J Freemont, Judith A Hoyland

**Affiliations:** 1 Tissue Injury and Repair Group, School of Clinical and Laboratory Sciences, Faculty of Human and Medical Sciences, University of Manchester, Stopford Building, Oxford Road, Manchester M13 9PT, UK

## Abstract

**Introduction:**

The aim of this study was to compare the effects of tumour necrosis factor-alpha (TNF-α) and interleukin-1-beta (IL-1β) on protease and catabolic cytokine and receptor gene expression in normal and degenerate human nucleus pulposus cells in alginate culture.

**Methods:**

Cells isolated from normal and degenerate nucleus pulposus regions of human intervertebral discs were cultured in alginate pellets and stimulated by the addition of 10 ng/mL TNF-α or IL-1β for 48 hours prior to RNA extraction. Quantitative real-time polymerase chain reaction was used to assess the effect of TNF-α or IL-β stimulation on the expression of matrix metalloproteinase (MMP)-3, -9 and -13, TNF-α, TNF receptor 1 (TNF-R1), TNF receptor 2 (TNF-R2), IL-1α, IL-1β, IL-1 receptor 1 (IL-1R1) and IL-1 receptor antagonist (IL-1Ra).

**Results:**

*MMP-3 *and *MMP-9 *gene expressions were upregulated to a greater level by IL-1β than TNF-α. *MMP-13 *was upregulated by each cytokine to a similar extent. *TNF-α *and *TNF-R2 *expressions were upregulated by both TNF-α and IL-β, whereas *TNF-R1 *expression was not significantly affected by either cytokine. *IL-1β *and *IL-1Ra *expressions were significantly upregulated by TNF-α, whereas *IL-1α *and *IL-1R1 *were unchanged.

**Conclusions:**

TNF-α does not induce *MMP *expression to the same degree as stimulation by IL-1β, but it does act to upregulate *IL-1β *expression as well as *TNF-α *and *TNF-R2*. The net result of this would be an increased inflammatory environment and accelerated degradation of the matrix. These results support the hypothesis that, while TNF-α may be an important initiating factor in matrix degeneration, IL-1β plays a greater role in established pathological degradation.

## Introduction

Disc degeneration is a major economic and social burden that affects large numbers of people. It is a major cause of back pain, which is one of the commonest causes of morbidity in the West. Within the UK, approximately 11 million people experience lower back pain for at least one week out of every month, and it is estimated to cost approximately £11 billion in lost production due to absence from work [1]. Despite this, the pathogenesis of degeneration is a complex process that is poorly understood.

The intervertebral disc (IVD) is a fibrocartilaginous tissue situated between the vertebrae of the spine. It provides stability and flexibility to the spinal column, allowing movement in all directions. The IVD is composed of a central gelatinous nucleus pulposus (NP), which provides the compressibility of the tissue, and a surrounding fibrous annulus fibrosus (AF). The NP is composed predominantly of the proteoglycan aggrecan and type II collagen and is highly hydrated, whereas the AF is made up of concentric lamellae of highly organised type I collagen fibres that provide the tensile strength and restrain the inner NP region. Molecular changes in degeneration include altered matrix synthesis, including a decrease in glycosaminoglycan production and an increase in collagen type I within the NP, and upregulation of matrix-degrading enzymes [2-5]. This results in an increase in matrix destruction, decrease in tissue hydration, increase in fissure formation and loss of disc height. These catabolic processes are thought to be mediated by soluble factors such as the pro-inflammatory cytokines interleukin-1-beta (IL-1β) and tumour necrosis factor-alpha (TNF-α) [6-9]. Histological studies by Bachmeier and colleagues [10,11] have shown TNF-α and its receptors to be expressed in normal IVD and upregulated with age and degeneration. Seguin and colleagues [12] have demonstrated in bovine cultures that TNF-α decreases expression of aggrecan and type II collagen genes and upregulates mRNA expression of matrix metalloproteinase (MMP)-1, -3 and -13 and ADAM-TS4 (a disintegrin and metalloproteinase with thrombospondin motif 4) and ADAM-TS5, resulting in a net catabolic response. Previous studies from this laboratory have investigated the expression of IL-1β and associated receptors in disc degeneration and shown that IL-1α, IL-1β, IL-1 receptor 1 (IL-1R1) and IL-1 receptor antagonist (IL-1Ra) are expressed by normal disc cells, with an upregulation of IL-1α, IL-1β and IL-1R1, but not the IL-1Ra, during degeneration [7].

Furthermore, we have shown that, while both IL-1 and TNF are expressed in IVD and upregulated with degeneration, degenerate IVDs show a much greater expression level of IL-1β than TNF-α and that, while the IL-1R1 was upregulated in degeneration, the TNF receptor 1 (TNF-R1) was not [8,13,14]. However, there have been few studies comparing the effects of IL-1β and TNF-α in adult human tissue or cells. A recent study from our laboratory investigated the effect of IL-1β or TNF-α or their antagonists on matrix-degrading activity from normal or degenerate cells as determined by *in situ *zymography [14]. The results indicated that in all cases the basal degradative activity of degenerate cells was greater than for normal cells and that this was not significantly affected by treatment with either exogenous IL-1β or TNF-α. However, the matrix-degrading activity in normal tissue was significantly upregulated by the addition of IL-1β, but not TNF-α. Furthermore, enzyme activity was inhibited in both normal and degenerate samples by the addition of IL-1Ra but unaffected by the application of anti-TNF-α. These results suggest that IL-1β, rather than TNF-α, may be more important in the regulation of matrix-degrading enzymes in IVD tissue, although the presence of TNF-α and TNF receptors suggests that this cytokine may have a role to play in IVD matrix regulation. Both cytokines have been shown to upregulate catabolic processes in articular cartilage and the IVD that lead directly to matrix degradation, and both TNF-α and IL-1β are thought to be pivotal to the cartilage destruction in arthritis [15,16]. This study compares the effect of TNF-α and IL-1β on catabolic gene expression that leads to matrix regulation and degradation in normal and degenerate NP cells isolated from human adult tissue and investigates how TNF-α and IL-1β may be involved in regulation of themselves and each other and their respective receptors.

## Materials and methods

### Tissue source

All human tissue was obtained in accordance with the Declaration of Helsinki, and ethical approval for these studies was obtained from the Trent Multi-centre Research Ethics Committee (reference 05/MRE04/3). Human IVD samples were obtained either from post-mortem samples or from surgery with the informed consent of patients or relatives. All samples were assessed histologically and graded for degeneration according to the method of Sive and colleagues [17]. Samples from three normal discs (histological grades 1 and 2; mean age 48 years, range 37 to 61 years) and three degenerate discs (histological grades 7, 9 and 10; mean age 58.3 years, range 35 to 79 years) were used for this study.

### Cell culture

Cells were isolated from the central NP region of the IVD by enzymatic digestion [7]; samples from each patient and from normal and degenerate discs were processed separately. Cells were cultured in standard medium – Dulbecco's modified Eagle's medium with glucose 4.5 g/L, GlutaMAX™ and pyruvate (Gibco, now part of Invitrogen Corporation, Paisley, UK) containing 50 μg/mL ascorbic acid, 250 ng/mL amphotericin, 100 U/mL penicillin and 100 μg/mL streptomycin (Invitrogen Corporation) and 10% (vol/vol) foetal calf serum (Invitrogen Corporation) – and expanded in monolayer. Culture media were changed every 3 days. Cells were grown in monolayer until they reached 70% to 80% confluence before passaging to expand cell numbers. Passage number was kept to a minimum as high numbers of passages have been reported to influence cell response. A passage number greater than 6 was shown to have a marked effect on disc cell behaviour (J.A. Hoyland, unpublished data); therefore, all cell samples were used at passage 4 or less.

### Culture of disc cells in alginate beads

Cells were re-suspended at a density of 4 × 10^6 ^cells/mL (equivalent to the cell density in the disc *in vivo *[18].) in 1.2% (wt/vol) low-viscosity sodium alginate in 0.15 M NaCl and cultured for 2 weeks prior to cytokine treatment to allow the cells to regain their native phenotype. We and others have previously shown that IVD cells expanded in monolayer will re-differentiate back to an *in vivo *phenotype if cultured in alginate beads [7,19]. Culture media were changed every 3 days. Following cytokine treatment, cells were recovered from alginate beads using dissolving buffer (55 mM sodium citrate, 30 mM EDTA [ethylenediaminetetraacetic acid], 0.15 M NaCl, pH 6.0) and then pelleted by centrifugation before RNA extraction.

### Treatment of disc cells with interleukin-1-beta or tumour necrosis factor-alpha

Standard culture media were replaced with 2 mL of media containing 10 ng/mL of either human recombinant IL-1β (AMS Biotechnology Ltd, Abingdon, UK) or human recombinant TNF-α (AMS Biotechnology Ltd). Cells were stimulated for 48 hours and maintained at 37°C in a humidified atmosphere containing 5% CO_2_. All treatments were carried out in triplicate. This protocol has previously been shown to be sufficient for IL-1β to significantly upregulate gene expression of *MMP-3 *and -*13 *and *ADAM-TS4 *[7] and for TNF-α to significantly upregulate several of the *ADAM-TS *family (A. Pockert, J.A. Hoyland, unpublished data).

### RNA extraction

Samples for RNA extraction were pelleted by centrifugation and the RNA was extracted using TRIzol™ and PureLink columns in accordance with the instructions of the manufacturer (Invitrogen Corporation). To prevent any DNA contamination of the RNA, the samples were treated with DNAse I solution (Qiagen Ltd., Crawley, UK), and RNA quality and quantity were determined using the Nanodrop^® ^ND-1000 Spectrophotometer (NanoDrop Technologies, Inc., now part of Thermo Fisher Scientific Inc., Waltham, MA, USA). cDNA was synthesised from 500 ng of RNA using Superscript™ II Reverse Transcriptase (Invitrogen Corporation) and random primers in accordance with the instructions of the manufacturer cDNA was stored at -20°C until required.

### Quantitative real-time polymerase chain reaction

Real-time primers and probes for *GAPDH *(*glyceraldehyde-3-phosphate dehydrogenase*), *TNF-α, TNF-R1, IL-1α, IL-1β, IL-1R1, IL-1Ra, MMP-3, -9, -13, aggrecan, collagen types I *and *II *and *SOX9 *were designed for TaqMan polymerase chain reaction (PCR) using Primer Express software (Applied Biosystems, Warrington, UK) based on the genomic sequences supplied in the GenBank database. Specificity for the primers and probes was confirmed by BLAST (Basic Local Alignment Search Tool) analysis. Primers and probe for *TNF receptor 2 (TNF-R2) *were purchased from Applied Biosystems as a pre-designed assay (PDAR). Sequences for primers and probes are presented in Table 1.

**Table 1:** Sequences of primers and probes used in quantitative real-time polymerase chain reaction analysis

Target	Forward primer	Reverse primer	Probe	Accession number
GAPDH	GCTGAACGGGAAGCTCACT	AGGTCAGGTCCACCACTGA	CCCCACTGCCAACGTG	[GenBank: NM_002046]
Aggrecan	CCGTGTGTCCAAGGAGAAGG	GGGTAGTTGGGCAGTGAGAC	CTGATAGGCACTGTTGAC	[GenBank: NM_001135]
Collagen type I	AGAACAGCGTGGCCTACATG	GCGCGGATCTCGATCTCG	CAGCAGACTGGCAAC	[GenBank: NM_000088]
Collagen type II	ATGGAGACTGGCGAGACTTG	GCTGCTCCACCAGTTCTTCTT	CCCAATCCAGCAAACG	[GenBank: NM_001844]
SOX9	GACTTCCGCGACGTGGAC	GTTGGGCGGCAGGTACTG	CGACGTCATCTCCAACAT	[GenBank: NM_000346]
IL-1α	AAGAGGGAAGTTTGCTTGATTAAGG	GAGGATCAAGACTTCTTTGTGCTC	ACCACTGTTCTCTTCTCTACCCTGCCC	[GenBank: NM_000575]
IL-1β	CGGCCACATTTGGTTCTAAGA	AGGGAAGCGGTTGCTCATC	ACCCTCTGTCATTCG	[GenBank: NM_000576]
IL-1 receptor I	ATTTCTGGCTTCTAGTCTGGT	AACGTGCCAGTGTGGAGTGA	ACTTGATTTCAGGTCAATAACGGTCCCC	[GenBank: NM_000877]
IL-1 receptor antagonist	CCTGCAGGGCCAAGCA	GCACCCAACATATACAGCATTCA	AGCCTCGCTCTTGGCAGGTACTCAGT	[GenBank: NM_173841]
TNF-α	CGAACATCCAACCTTCCCAAAC	TGGTGGTCTTGTTGCTTAAAGTTC	CCAATCCCTTTATTACCC	[GenBank: NM_000594]
TNF receptor 1	AATTCTGGCTTCTAGTCTGGT	AACGTGCCAGTGTGGAGTGA	TTCAGTCCCACTCCAGGCTTCACCC	[GenBank: NM_001065]
TNF receptor 2	PDAR	PDAR	PDAR	
MMP-3	TGAAGAGTCTTCCAATCCTACTGTTG	CTAGATATTTCTGAACAAGGTTCATGC	TTTGCTCAGCCTATCCAT	[GenBank: NM_002422]
MMP-9	CCCGGAGTGAGTTGAACCA	CAGGACGGGAGCCCTAGTC	TACGTGACCTATGACATC	[GenBank: NM_004994]
MMP-13	CCCCAGGCATCACCATTCAAG	GACAAATCATCTTCATCACCACCAC	CTGCCTTCCTCTTC	[GenBank: NM_002427]

GAPDH, glyceraldehyde-3-phosphate dehydrogenase; IL-1, interleukin-1; MMP, matrix metalloproteinase; PDAR, pre-designed assay; TNF, tumour necrosis factor.

All PCRs were set up in triplicate using a final volume of 25 μL (2.5 μL cDNA) and Universal Taq mastermix (Applied Biosystems). All primers were used at 900 nM, and probes were used at 250 nM. Reactions were carried out and analysed by an ABI Prism 7700 Detection System.

Initial analysis was performed using the 7000 System Sequence Detection Software package (Applied Biosystems) as a relative quantification study using *GAPDH *as the endogenous control gene and using the automatic settings to set baseline and threshold values. The data were then exported into Excel (Microsoft Corporation, Redmond, WA, USA) for further analysis. Data were analysed using the 2^-ΔΔCt ^method using the housekeeping gene *GAPDH *and untreated controls for normalisation [7].

### Statistics

All samples were tested for normality using the Shapiro-Wilk test. As all data were non-parametric, they were then analysed by the Mann-Whitney *U *test.

## Results

### Effect of cytokines on matrix-degrading enzymes

There was no difference in the basal levels of gene expression for *MMP-3, -9 *or *-13 *between untreated normal and degenerate NP cells (data not shown).

### Tumour necrosis factor-alpha

**Figure 1:**
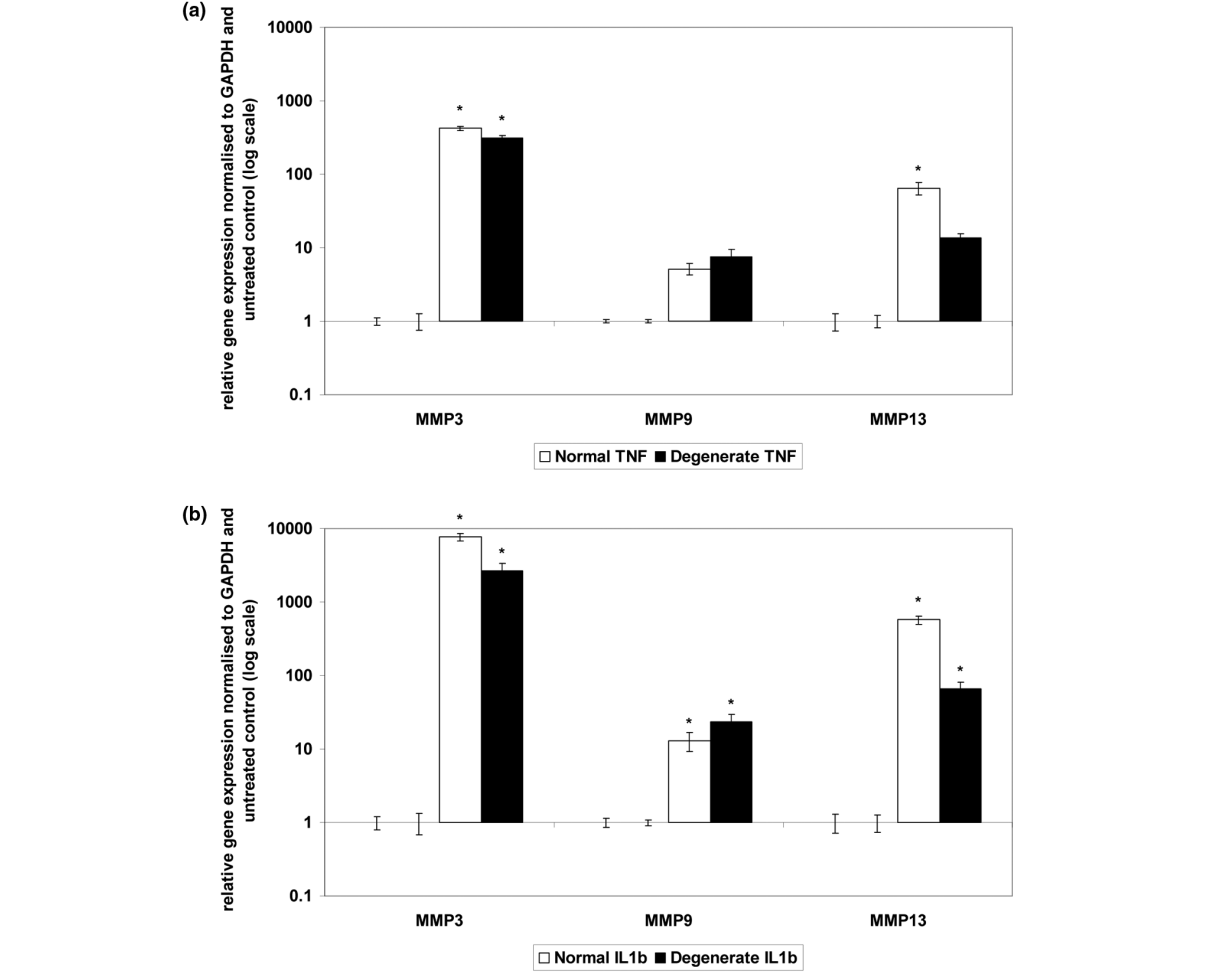
Effect of cytokine stimulation on matrix metalloproteinase (MMP) gene expression in normal or degenerate nucleus pulposus (NP) cells. Normal or degenerate NP cells were cultured in alginate pellets and stimulated by the addition of 10 ng/mL tumour necrosis factor-alpha (TNF-α) **(a) **or interleukin-1-beta (IL-1β) **(b)**. Quantitative real-time polymerase chain reaction was used to analyse the effect of cytokine stimulation on gene expressions of *MMP-3, -9 *and -*13*. All samples are relative to the housekeeping gene *GAPDH *(glyceraldehyde-3-phosphate dehydrogenase) and normalised back to untreated controls. Results are given as mean ± standard error of the mean (n = 3). *P ≤ 0.05 when compared with untreated controls.

The addition of recombinant TNF-α for 48 hours resulted in a significant upregulation of *MMP-3 *mRNA in both normal and degenerate NP cells (*P *= 0.05) and *MMP-13 *gene expression in normal cells (*P *= 0.05) (Figure 1a). There was no significant change in gene expression for *MMP-9 *in normal or degenerate cells or for *MMP-13 *in degenerate cells (Figure 1a).

### Interleukin-1-beta

The addition of recombinant IL-1β for 48 hours resulted in a significant upregulation of mRNA of *MMP-3, -9 *and *-13 *in both normal and degenerate NP cells (*P *= 0.05) (Figure 1b). The upregulation of *MMP-9 *gene expression was significantly greater in degenerate cells compared with normal NP cells (*P *= 0.05), and the upregulation of *MMP-13 *was significantly greater in normal than degenerate cells (*P *= 0.05). There was no significant difference between the upregulation of *MMP-3 *in normal and degenerate NP samples.

The upregulation of *MMP-3 *mRNA was significantly greater by recombinant IL-1β than TNF-α in both normal and degenerate cells (normal: 7,695-fold and 420-fold, respectively; degenerate: 2,663-fold and 315-fold, respectively; *P *= 0.05). IL-1β had a significantly greater effect than TNF-α on *MMP-9 *gene upregulation in degenerate NP cells (23.5-fold and 7.6-fold, respectively; *P *= 0.05), although the difference between IL-1β-associated *MMP-9 *upregulation and TNF-α-stimulated *MMP-9 *gene upregulation in normal NP cells was not significant. There was no significant difference between the effect of IL-β and TNF-α on *MMP-13 *gene upregulation in normal and degenerate NP cells.

### Effect of tumour necrosis factor-alpha on matrix gene expression

The basal gene expression for the *collagen type I *gene was significantly higher in the degenerate cells than in the normal NP cells (*P *= 0.05). There was no significant difference in the basal gene expression levels between normal and degenerate cells for any of the other matrix genes examined (data not shown).

**Figure 2:**
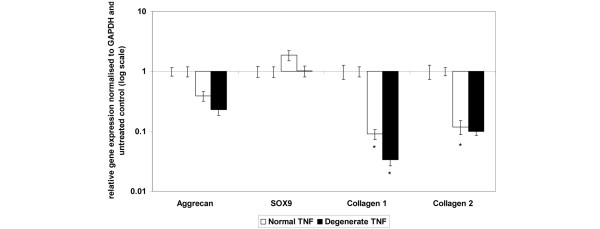
Effect of tumour necrosis factor-alpha (TNF-α) stimulation on matrix gene expression in normal or degenerate nucleus pulposus (NP) cells. Normal or degenerate NP cells were cultured in alginate pellets and stimulated by the addition of 10 ng/mL TNF-α. Quantitative real-time polymerase chain reaction was used to analyse the effect of TNF-α stimulation on gene expression of *collagen type I (c1)*, *collagen type II (c2)*, *SOX9 (s9) *and *aggrecan (agg)*. All samples are relative to the housekeeping gene *GAPDH *(glyceraldehyde-3-phosphate dehydrogenase) and normalised back to untreated controls. Results are given as mean ± standard error of the mean (n = 3). *P ≤ 0.05 when compared with untreated controls.

The addition of recombinant TNF-α for 48 hours significantly downregulated *collagen type I *gene expression in both normal and degenerate NP samples (Figure 2), with the downregulation in degenerate samples being significantly greater than for normal samples (*P *= 0.05). *Type II collagen *mRNA was significantly downregulated in normal NP cells following the addition of TNF-α (*P *= 0.05), but expression was not significantly changed in degenerate cells (Figure 2). There was no significant change in the gene expression of *aggrecan *or *SOX9 *in either normal or degenerate NP cells following TNF-α stimulation (Figure 2).

### Effect of inflammatory cytokines on expression of tumour necrosis factor-alpha and tumour necrosis factor receptors

There was no difference in the basal expression levels of the inflammatory cytokine genes or their receptors in normal and degenerate untreated NP cells (data not shown).

### Tumour necrosis factor-alpha

**Figure 3:**
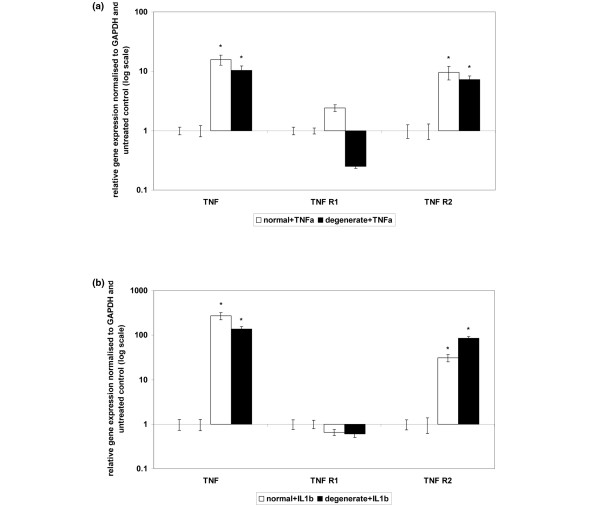
Effect of cytokine stimulation on tumour necrosis factor-alpha (TNF-α) and TNF receptor gene expression in normal or degenerate nucleus pulposus (NP) cells. Normal or degenerate NP cells were cultured in alginate pellets and stimulated by the addition of 10 ng/mL TNF-α **(a) **or IL-1β **(b)**. Quantitative real-time polymerase chain reaction was used to analyse the effect of cytokine stimulation on *TNF-α, TNF receptor 1 (TNF-R1) *and *TNF receptor 2 (TNF-R2) *gene expression All samples are relative to the housekeeping gene *GAPDH *(glyceraldehyde-3-phosphate dehydrogenase) and normalised back to untreated controls. Results are given as mean ± standard error of the mean (n = 3). *P ≤ 0.05 when compared with untreated controls.

The addition of recombinant TNF-α for 48 hours resulted in a significant upregulation of *TNF-α *and *TNF-R2 *genes in both normal and degenerate NP cells (Figure 3a). *TNF-R1 *mRNA was upregulated by TNF-α in normal NP cells but downregulated in degenerate samples (Figure 3a). Neither of these changes in mRNA expression was statistically significant, although the difference between the upregulation in normal cells and the downregulation in degenerate cells did reach significance (*P *= 0.05). *TNF-R2 *mRNA was upregulated to a greater extent in normal than in degenerate samples (*P *= 0.05).

### Interleukin-1-beta

The addition of exogenous IL-1β for 48 hours resulted in a statistically significant upregulation of *TNF-α *and *TNF-R2 *genes in both normal and degenerate samples (*P *= 0.05) (Figure 3b). The upregulation of *TNF-α *mRNA was significantly greater in normal NP cells than degenerate samples (*P *= 0.05), whereas the *TNF-R2 *gene was upregulated to a greater extent in degenerate cells than normal NP samples (*P *= 0.05). *TNF-R1 *gene expression was not significantly affected by the addition of IL-1β in either normal or degenerate samples.

### Effect of tumour necrosis factor-alpha on expression of interleukin-1 and interleukin-1 receptors

**Figure 4:**
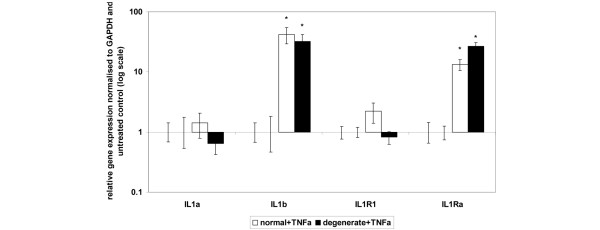
Effect of tumour necrosis factor-alpha (TNF-α) stimulation on interleukin-1 (IL-1) and IL-1 receptor gene expression in normal or degenerate nucleus pulposus (NP) cells. Normal or degenerate NP cells were cultured in alginate pellets and stimulated by the addition of 10 ng/mL IL-1β. Quantitative real-time polymerase chain reaction was used to analyse the effect of cytokine stimulation on gene expressions of *IL-1α, IL-1β, IL-1-receptor 1 (IL-1R1) *and *IL-1-receptor antagonist (IL-1Ra)*. All samples are relative to the housekeeping gene *GAPDH *(glyceraldehyde-3-phosphate dehydrogenase) and normalised back to untreated controls. Results are given as mean ± standard error of the mean (n = 3). *P ≤ 0.05 when compared with untreated controls.

The addition of recombinant TNF-α for 48 hours resulted in a significant upregulation of *IL-1β *and *IL-1Ra *genes in both normal and degenerate NP samples (*P *= 0.05) (Figure 4). There was no significant difference between the degree of upregulation of either *IL-1β *or *IL-1Ra *genes in normal and degenerate cells. *IL-1α *and *IL-1R1 *gene expressions were not significantly altered in either normal or degenerate NP cells by the action of TNF-α.

## Discussion

This study investigates the effect of the pro-inflammatory cytokines IL-1β and TNF-α on catabolic gene expression in normal and degenerate IVD cells. Previous studies from our laboratory have shown that, in the human, MMP-1, -3, -7 and -13 are upregulated with increasing degeneration [2,7] and that stimulation of cells in culture with IL-1β results in an upregulation of MMP-3 and -13 as well as ADAM-TS4 and a downregulation of aggrecan and collagen types I and II [7]. In this study, we have expanded the investigation to include the effect of TNF-α on matrix and MMP gene expression in normal and degenerate NP cells as well as the effect of IL-1β and TNF-α on cytokine gene expression and the expression of the associated receptors and endogenous antagonists. There was no difference in the basal levels for expression of each of the genes studied between normal and degenerate untreated cells, with the exception of *type I collagen*; thus, any changes identified were due to the stimulatory or inhibitory effect of the recombinant cytokines added to the cultures rather than any difference in the initial levels. For *type I collagen*, there was a higher basal level in degenerate cells when compared with normal NP cells. This is consistent with the changes identified in the degeneration of the IVD, which include an upregulation in type I collagen protein in the NP region [20].

The results of this study show that TNF-α downregulates the matrix genes *collagen types I *and *II *and *aggrecan*. This is consistent with studies on bovine tissue which have shown that TNF-α downregulates *aggrecan *and *collagen type II *[12] and previous studies from our laboratory which have shown that IL-1β significantly downregulates *collagen types I *and *II *and *aggrecan *[7]. TNF-α had no effect on the matrix-associated transcription factor *SOX9*, a finding that is consistent with previous findings from our laboratory which found that IL-1β did not significantly affect *SOX9 *gene expression, although *SOX6 *was significantly decreased by IL-1β stimulation [7].

MMPs can be largely grouped in three classes by the substrates they degrade: collagenases (including MMP-1 and -13) that break down fibrillar collagens, gelatinases (MMP-2 and -9) that act on denatured collagens and collagen types IV and V, and stromelysins (including MMP-3) that degrade predominantly non-collagenous proteins such as proteoglycans and fibronectin and can activate the pro-collagenases. Having previously demonstrated that IL-1β downregulates *MMP-3 *and -*13 *and that increased expression of IL-1Ra can reduce the activity of MMP-1, -3, -7 and -13 in tissue explants [6], we investigated the effect of TNF-α stimulation on expression of MMPs from each of these groups and compared it directly with IL-1β stimulation. Whereas there have been several studies that have shown that both IL-1β and TNF-α upregulate metalloproteinase activity in cartilage, surprisingly little has been done to compare the effects of the two cytokines on matrix-degrading activity, with none in human studies. Richardson and Dodge [21] showed that TNF-α and IL-1β both upregulated *MMP*-*1, -3 *and -*13 *in equine cartilage by up to 100-fold and that *type II collagen *and *aggrecan link-protein *mRNAs were also decreased, whereas Lefebvre and colleagues [22] demonstrated that IL-1β was more effective than TNF-α in upregulating gelatinase activity, specifically MMP-9, in rabbit articular chondrocytes. This study has shown that, in regard to the three MMPs investigated, TNF-α and IL-1β had the greatest effect on the gene expression of *MMP-3*, followed by *MMP-13*, then *MMP-9*. *MMP-3 *was upregulated to a greater extent by IL-1β than TNF-α in both normal and degenerate cells, whereas *MMP-9 *was upregulated to a greater extent by IL-1β than TNF-α in degenerate cells. In degenerative cartilage diseases such as osteoarthritis, degradation of the proteoglycans has been shown to precede breakdown of the collagenous molecules [23], so a greater initial upregulation of stromelysins may be significant in this regard. Furthermore, MMP-3 can activate MMP-9 and -13 (as well as other members of the MMP family), thereby increasing general metalloproteinase activity without the need to upregulate mRNA expression. This activation would result in not only a greater degradation of the non-collagenous matrix molecules such as proteoglycans, but potentially an increased activation of the collagenases that degrade the fibrillar collagens. MMP-13 has a particular affinity for type II collagen, a matrix molecule that is abundant within the NP, whereas MMP-9 cleaves denatured collagens.

Stimulation of NP cells by the addition of TNF-α resulted in the upregulation of *TNF-α *and *TNF-R2 *in both normal and degenerate cells, although the levels of *TNF-R1 *remained unchanged. This finding is in keeping with previous studies from our group which have shown that *TNF-α *and *TNF-R1 *are expressed by the IVD, but the *TNF-R1 *expression is not increased in degeneration [8]. It is not consistent with the findings of Bachmeier and colleagues [10], who found that, while TNF-R1 expression increased from approximately 20% in the 18-to-30 age group to approximately 35% in the 31-to-60 age group and then fell again to approximately 25% in the over-60 age group, expressions of TNF-R2 did not greatly differ among the various age groups studied. These studies investigated the protein expression of these receptors rather than gene expression and separated the groups according to age rather than degeneration, which may explain the discrepancy between the studies.

TNF-α can bind to and signal through either TNF-R1 or TNF-R2 receptors. Studies by Alsalameh and colleagues [24] on synovial fibroblasts from rheumatoid arthritis patients and osteoarthritis patients have indicated that there is a differential expression of the two TNF receptors in these cells and that, while both receptors can mediate the effect of TNF-α on TIMP1 expression, PGE_2 _(prostaglandin E_2_) IL-6 and MMP-1 regulations are mediated exclusively via TNF-R1, suggesting that, although the expression of TNF-R1 does not change with degeneration in the IVD, signalling through this receptor is critical for upregulation of degradative processes. TNF-α has been shown to upregulate *MMP-9 *gene expression in malignant carcinoma via TNF-R1 [25], and TNF-R1 but not TNF-R2 has been shown to be important in inflammatory responses involving IL-1β and MMP-3 and -9 in murine brain injury [26]. This suggests that, although *TNF-R1 *levels remain unchanged and *TNF-R2 *gene expression is increased, it is signalling through TNF-R1 that is important in upregulating metalloproteinases and inflammatory cytokines such as IL-1β and IL-6. However, studies on collagen-induced arthritis in *TNF-R1 *knockout mice have shown that the lack of TNF-R1 does not affect the incidence or severity of collagen-induced arthritis in the joints [27]. Repeated injections of TNF-α into the joints of TNF-R1 knockout mice enhanced the development of arthritis if such injections were administered during the early phase of arthritis induction but had little effect once the arthritis was established, indicating that TNF-R2 can be involved in the onset of cartilage degradation and degeneration [27]. The precise significance of the upregulation of *TNF-R2 *by TNF-α in NP cells remains to be elucidated.

Our data demonstrate that TNF-α also upregulates *IL-1β *and *IL-1Ra *genes, but not *IL-1α *or *IL-1R1*, whereas IL-1β upregulates *TNF-α *and *TNF-R2*. We have also previously shown that IL-1β upregulates itself and IL-1α whilst downregulating IL-1R1 and having no effect on the expression of IL-1Ra in IVD [7]. The net result of this cascade will be to increase the pro-inflammatory cytokine expression within the tissue. It has been suggested that TNF-α stimulates and drives the IL-1 production in cartilage [28]. This hypothesis is supported by antibody treatments of animal models of collagen-induced arthritis, in which anti-TNF-α reduces cartilage destruction, but is most effective at early stages of the disease [29,30]. Treatment with anti-IL-1 also is highly efficient at reducing cartilage destruction and continues to do so even when the disease is well established [29,31,32]. However, it is unclear whether this is the case in the IVD and how important TNF-α is to human IVD degeneration.

One possibility is that TNF-α upregulates IL-1β, which then upregulates itself and IL-1α and further upregulates TNF-α. This would result in an increase in matrix-degrading activity and accelerating degeneration and would be consistent with the suggestion that TNF-α is involved in the early onset of degeneration, stimulating and driving the IL-1β signalling, as is thought to occur in articular cartilage and osteoarthritis. If TNF-α is involved only as an initiating factor in the early onset of degradation in the IVD and then becomes unimportant as the IL-1β degradative mechanisms take over (as has been suggested in cartilage), it would also explain why antagonists to IL-1 signalling, but not TNF, would inhibit matrix-degrading activity in established degradation, as shown in our *in situ *zymography studies [14]. However, to be able to determine the precise role and importance of TNF-α in the initiation of IVD degeneration, many more studies are required.

## Conclusions

In this study, we have shown that TNF-α downregulates matrix molecule genes at the same time that it upregulates matrix molecule-degrading genes within the normal and degenerate NP. TNF-α does not induce *MMP *gene expression to the same degree as stimulation by IL-1β, but it does act to upregulate *IL-1β *gene expression as well as *TNF-α *and *TNF-R2 *mRNA. The net result of this would be an increased inflammatory environment and accelerated degradation of the matrix. These results are consistent with the hypothesis that, while TNF-α may be effective at an early stage of degeneration, IL-1β is a more potent stimulus as degeneration becomes prolonged and plays a greater role in established pathological degradation.

## Abbreviations

ADAM-TS: a disintegrin and metalloproteinase with thrombospondin motif; AF: annulus fibrosus; GAPDH: glyceraldehyde-3-phosphate dehydrogenase; IL: interleukin; IL-1R: interleukin-1 receptor; IL-1Ra: interleukin-1 receptor antagonist; IVD: intervertebral disc; MMP: matrix metalloproteinase; NP: nucleus pulposus; PCR: polymerase chain reaction; TNF: tumour necrosis factor; TNF-R: tumour necrosis factor receptor.

## Competing interests

The authors declare that they have no competing interests.

## Authors' contributions

SJM-S carried out a proportion of the experimental work, supervised PWC, analysed the data and drafted the manuscript. PWC carried out the remainder of the experimental work. AJF helped to secure funding and contributed to the preparation of the final manuscript. JAH conceived and designed the study, secured funding and co-wrote the manuscript. All authors read and approved the final manuscript.
